# *STIR-Net*: Deep Spatial-Temporal Image Restoration Net for Radiation Reduction in CT Perfusion

**DOI:** 10.3389/fneur.2019.00647

**Published:** 2019-06-26

**Authors:** Yao Xiao, Peng Liu, Yun Liang, Skylar Stolte, Pina Sanelli, Ajay Gupta, Jana Ivanidze, Ruogu Fang

**Affiliations:** ^1^J. Crayton Pruitt Family Department of Biomedical Engineering, University of Florida, Gainesville, FL, United States; ^2^Department of Radiology, Weill Cornell Medical College, New York, NY, United States; ^3^Imaging Clinical Effectiveness and Outcomes Research, Department of Radiology, Northwell Health, Manhasset, NY, United States; ^4^Donald and Barbara Zucker School of Medicine at Hofstra/Northwell, Hempstead, NY, United States; ^5^Center for Health Innovations and Outcomes Research, Feinstein Institute for Medical Research, Manhasset, NY, United States

**Keywords:** CT perfusion image, radiation reduction, image restoration, deep learning, brain hemodynamics

## Abstract

Computed Tomography Perfusion (CTP) imaging is a cost-effective and fast approach to provide diagnostic images for acute stroke treatment. Its cine scanning mode allows the visualization of anatomic brain structures and blood flow; however, it requires contrast agent injection and continuous CT scanning over an extended time. In fact, the accumulative radiation dose to patients will increase health risks such as skin irritation, hair loss, cataract formation, and even cancer. Solutions for reducing radiation exposure include reducing the tube current and/or shortening the X-ray radiation exposure time. However, images scanned at lower tube currents are usually accompanied by higher levels of noise and artifacts. On the other hand, shorter X-ray radiation exposure time with longer scanning intervals will lead to image information that is insufficient to capture the blood flow dynamics between frames. Thus, it is critical for us to seek a solution that can preserve the image quality when the tube current and the temporal frequency are both low. We propose STIR-Net in this paper, an end-to-end spatial-temporal convolutional neural network structure, which exploits multi-directional automatic feature extraction and image reconstruction schema to recover high-quality CT slices effectively. With the inputs of low-dose and low-resolution patches at different cross-sections of the spatio-temporal data, STIR-Net blends the features from both spatial and temporal domains to reconstruct high-quality CT volumes. In this study, we finalize extensive experiments to appraise the image restoration performance at different levels of tube current and spatial and temporal resolution scales.The results demonstrate the capability of our STIR-Net to restore high-quality scans at as low as 11% of absorbed radiation dose of the current imaging protocol, yielding an average of 10% improvement for perfusion maps compared to the patch-based log likelihood method.

## 1. Introduction

Acute stroke has high mortality and severe long-term disability rates worldwide. In the United States, more than 795,000 people have a stroke annually, and about 140,000 of them lose their lives, accounting for 5% of all deaths (1). Someone develops a stroke approximately every 40 s, and nearly every 4 min, someone loses he or her life because of stroke. Stroke can occur at any age, and it increases in likelihood with age. In 2009, two-thirds of people who had been hospitalized for stroke were older than 65 years old (2). The estimated cost related to stroke in the United States is about 34 billion dollars each year (3).

Acute stroke is an emergency, and successful patient outcomes require accurate diagnosis and prompt treatment. It is critical for someone to receive treatments for stroke within three hours from when he or she presents initial symptoms, as the disability rate measured three months after the stroke is generally high in those who did not receive timely treatments (4). There are two types of stroke: hemorrhagic and ischemic stroke. Hemorrhagic stroke occurs when a fragile blood vessel ruptures, while ischemic stroke is caused by thrombosis or embolism. Due to different etiologies and therapies, it is essential for patients to get timely diagnoses and treatments.

Computed Tomography (CT) scanning is a widely used imaging modality for rapid and detailed evaluation of the brain and cerebral vasculature; it is particularly valuable in the triage of acute stroke patients. CT can provide a rapid diagnosis of ischemic or hemorrhage stroke. It is clinically meaningful as rapid diagnosis enables clinicians to initiate optimized treatment for each of these two major categories of stroke. Patients with ischemic stroke often benefit from further characterization of brain tissue hemodynamics, and as such, often go through CT Perfusion (CTP) for further diagnosis and to guide treatment planning such as thrombolytic therapy. As CTP imaging can promptly offer an active view of cerebrovascular physiology, doctors can acquire CTP to evaluate cerebral blood flow status.

Obtaining a comprehensive visualization of blood flow dynamics and a clear brain anatomic structure requires contrast dose injection and repeated CT scanning. Under the acute stroke protocol, X-ray radiation from a 40-s CTP scan is comparable to a year's worth of radiation exposure from natural surroundings (5, 6). The CTP/CT Angiography (CTA) data acquisition process on a whole brain has a mean dose level of 6.8 mSv (7), which is two times more than that from natural background radiation sources; in comparison, the annual radiation exposure from the natural background is around 2.4 mSv (8). Moreover, repetitively scanning brain regions leads to accumulative radiation exposure to patients that may increase health risks such as skin irritation/erythema, hair loss/epilation (9), cataract formation (10), and even the induction of cancer (11, 12). In the US, about 80 million CT scans are performed annually. Therefore, seeking solutions to reduce the radiation dose that is associated with CT scans draws many researchers' attention.

Many researchers have attempted to seek practical solutions for radiation dose reduction in CT imaging. Solutions for reducing radiation exposure include two primary directions: optimizing CT systems and reducing contrast dose. Typical optimization of CT systems comprises shortening temporal sampling frequency and reducing radiation sources such as the tube current/voltage and the number of beams and receptors. However, a simple reduction by the methods above will increase image noise and artifacts. In order to reduce CTP radiation exposure and maintain high diagnostic image quality, we integrate a deep learning approach with CT imaging to carry out this study.

In this paper, we propose an end-to-end Spatial-Temporal Image Restoration Net (STIR-Net) for CTP image restoration. This structure consists of two main components: Super-Resolution Denoising Nets (SRDNs) and a multi-directional conjunction layer which addresses image super-resolution (SR) and denoising in both spatial and temporal cross-sections. The contributions of this work are five-fold:

1. SRDN's patch representation layer extracts features from both the spatial and temporal dimensions of the CTP volume as cross-sections, which allows our model to present spatial-temporal details at the same time.
2. SRDN has the ability to perform image SR and denoising individually and simultaneously. It also can handle multi-level noise and multi-scale resolution and sampling.
3. We integrate multiple SRDNs based on different cross-sections into a multi-directional network, which can boost the performance further than individual cross-sections.
4. The results of the experiments demonstrate the effectiveness of STIR in the recovery of low radiation dose CTP images. STIR-Net can provide practical solutions for radiation dose reduction from three aspects (low tube current, decreased temporal sampling rate, and poor spatial resolution) with comparable image quality to the standard dose protocol.
5. We also provide the comparisons of Cerebral Blood Flow (CBF) and Cerebral Blood Volume (CBV), these maps attest that our proposed method can provide comparable results to the existing methods.

It is important to point out that no work has addressed low tube current, decreased temporal sampling rate, and poor spatial resolution simultaneously with a single deep learning structure. Through extensive experiments, our results demonstrate that STIR-Net has the capability of image restoration from these three types of data limitations simultaneously. Compared to low-dose scans using conventional methods, our network yields an average of 21% improvement of peak signal-to-noise ratio (PSNR) at around 21% to 42% low tube currents for the CTP sequences and an average of 10% improvement for the calculated perfusion maps. Hence, STIR-Net is a promising method for reducing radiation exposure in CTP imaging.

## 2. Related Work

It is necessary to develop low-dose CTP protocols to reduce the risks associated with excessive X-ray radiation exposure. Different acquisition parameters such as tube current, temporal sampling frequency, and the spatial resolution are meticulously related to the quality of the reconstructed CTP images, especially for generating perfusion maps that will be directly used by doctors to make treatment decisions. Related work includes radiation dose reduction approaches with respect to image processing strategies, deep learning approaches, image SR methods, and denoising methods. The previous work of our spatio-temporal architecture is introduced at the end of this section.

### 2.1. Radiation Dose Reduction Approaches

Radiation dose reduction approaches include reducing tube current, temporal sampling frequency, and beam number. There is a linear relationship between radiation dose and the tube current. For example, lowering 50% of the tube current will lead to a 50% reduction in radiation dose. However, image noise and the square root of tube current have an inverse proportional relationship. Simply reducing the tube current will deteriorate the CTP image quality with increased noise and artifacts. Current simulation studies demonstrate the possibility and the effectiveness of maintaining image quality at reduced tube current (13, 14). Reducing temporal sampling frequency is the same as the increment of time intervals between acquiring two CTP slices in the same CT study. Similar to the decrement of the tube current, the reduction in temporal sampling frequency will reduce radiation correspondingly, as the total amount of scanning period is fixed and the time interval has been increased. However, current research (15–17) shows that the reductions in sampling interval yield little advantages when the time intervals are greater than 1 s.

### 2.2. Image-Based Radiation Dose Reduction Approaches

Acquiring CT scans at low-dose and long scanning intervals will result in noisy and low-resolution (LR) images, with insufficient hemodynamic information. It is important to obtain higher quality CT images from limited data. Therefore, we address this problem of CT radiation reduction as image-based dose reduction. Recent work shows that an image-based dose reduction approach is a promising way for CT radiation reduction. For example in Yu et al. (18), a study of pediatric abdomen, pelvis, and chest CT examinations demonstrate that a 50% dose reduction can still maintain diagnostic quality. The image-based approaches include iterative reconstruction algorithm, sparse representation and dictionary learning, and example-based restoration methods. We review the relevant work as follows.

The iterative reconstruction (IR) algorithm is a promising approach for dose reduction. It produces a set of synthesized projections by meticulously modeling the data acquisition process in CT imaging. For example, adaptive statistical iterative reconstruction (ASIR) algorithm (19) was the first IR algorithm to be used in the clinic. By modeling the noise distribution of the acquired data, ASIR can provide clinically acceptable image quality at reduced doses. Many CT systems apply ASIR as an assuring radiation dose reduction approach because it can reduce image noise and provide dose-reduced clinical images with preserved diagnostic value (20). Another IR algorithm is called model-based iterative reconstruction, which is more complicated and accurate than ASIR, as it models photons and system optics jointly.

Sparse representation and dictionary learning describe data as linear combinations of several fundamental elements from a predefined collection called a dictionary. In the computer vision and medical image analysis domains, sparse representation and dictionary learning have shown promising results in various image restoration applications. Such applications include sparsity-based simultaneous denoising and interpolation (21) for optical coherence tomography images reconstruction, dictionary learning with group sparsity and graph regularization (22) for medical image denoising and fusion, and (23) for magnetic resonance image reconstruction.

The example-based restoration approach is another popular method for image restoration. It extracts and stores patch pairs from both low-quality images and high-quality images in a database as prior knowledge. At the restoring phase, it learns a model that can synthesize high-quality images by searching the best-matched paired patches. Applications in image restoration (24–26) show the promising performance by using prior knowledge.

### 2.3. Deep Learning

In recent years, deep learning methods have emerged in various computer vision tasks, including image classification (27) and object detection (28), and have dramatically improved the performance of these systems. These approaches have also achieved significant improvement in image restoration (29, 30), super-resolution (31), and optical flow (32). The reason for the significant performance is due to the advanced modeling capabilities of the deep structure and the corresponding non-linearity combined with discriminative learning on large datasets.

Convolutional Neural Network (CNN), as one of the most renowned deep learning architectures, shows promising results for image-based problems. CNN structures are usually composed of several convolutional layers with activation layers, followed by one or more fully connected layers. The CNN architecture design utilizes image structures via local connections, weights sharing, and non-linearity. Another benefit of CNN is that they are easier to train and have fewer parameters than fully connected networks with the same number of hidden units. CNN structures allow automatic feature extraction and learning from limited information to reconstruct high-quality images.

### 2.4. Image Super-Resolution

Image super-resolution aims at restoring HR images from the observed LR images. SR methods use different portions of LR images, or separate images, to approximate the HR image. There are two types of SR algorithms: frequency domain-based and spatial domain-based. Initially, SR methods were mostly for problems in the frequency domain (33, 34). Algorithms addressed in the frequency domain using a simple theoretical basis for observing the relationships between HR and LR images. Though these algorithms show high computational efficiency, they are limited due to sensitivity to model errors and difficulty in managing complex motion models. Algorithms for the spatial domain then became the main trend by overcoming the drawbacks of the frequency domain algorithms (35). Predominate spatial domain methods include non-uniform interpolation (36), iterative back-projection (37), projection onto convex sets (38), regularized methods (39), and a number of hybrid algorithms (40).

Deep learning is a popular approach for image SR problems, and it has achieved significant performance (31, 41–43). However, most SR frameworks focus on 2D images, as involving the temporal dimension is more challenging, especially in CTP imaging. In this work, we propose to overcome the difficulties involving spatial dimension and to prove the feasibility of our framework in cerebral CTP image restoration.

### 2.5. Image Denoising

Image denoising tasks aim at recovering a clean image from an observed noisy image, whereas the observed image is intruded by additive Gaussian noise. One of the main challenges for image denoising is to accurately identify the noise and remove it from the observed image. Based on the image properties being used, existing methods can be classified as prior-based (44), sparse coding based (25), low-rank-based (45), filter-based (46), and deep learning based (47, 48). The filter-based approach (46) methods are classical and fundamental, and many subsequent studies are developed from it (49).

Numerous works have reconstructed clean CT images that can preserve the image quality of perfusion maps successfully; these works include methods such as bilateral filtering, non-local mean (50), nonlinear diffusion filter (51), and wavelet-based methods (52). The oscillatory nature of the truncated singular value decomposition (TSVD)-based method has initiated research that incorporates different regularization methods to stabilize the deconvolution. This research has shown varying degrees of success in stabilizing the residue functions by enforcing both temporal and spatial regularization on the residue function (53, 54). However, prior studies have focused exclusively on regularizing the noisy low-dose CTP, without considering the corpus of high-dose CTP data and the multi-dimensional data properties of CT images.

Recently, deep learning based methods (47, 48) have shown many advantages in learning the mapping of the observed low-quality images to the high-quality ones. These methods use CNN models that are trained on tens of thousands of samples; however, paired training data is usually scarce in the medical field. Hence, an effective learning based model is desired. In this work, we utilize data extracted from different cross-sections of the CTP volume to achieve better performance in image SR and denoising. The experiment result shows that the proposed network can handle various noise and image degradation levels.

### 2.6. Spatial-Temporal Architecture

In our previous work, we proposed Spatio-Temporal Architecture for Super-Resolution (STAR) (55) for low-dose CTP image super-resolution. It is an end-to-end spatio-temporal architecture that preserves image quality at reduced scanning time and radiation that has been reduced to one-third of its original level. This is an image-based dose reduction approach that focuses on super-resolution only. STAR is inspired by the work in Kim et al. (31) and is extended to three-dimensional volumes by conjoining multiple cross-sections. Through this work, we found that features extracted from both spatial and temporal directions are helpful to improve SR performance. The integration of multiple single-directional networks (SDNs) can boost the performance of SR for the spatio-temporal CTP data. The experimental results show that the proposed basic model of SDN improves both spatial and temporal resolution, while the multi-directional conjoint network further enhances the SR results—comparing favorably with only temporal or only spatial SR. However, this work only addresses low spatial and temporal resolution; it misses the important noise issue in low dose CTP.

In this paper, we propose STIR-Net, an end-to-end spatial-temporal image restoration net for CTP radiation reduction. We compose and integrate several SRDNs instead of SDNs at different cross-sections for both image super-resolution and denoising simultaneously. The STIR-Net structure is explained in section 3. In section 4, we provide the experiment platform setup and describe the data acquisition method and the preprocessing procedures. In section 5, we detail the experiments and results. Finally, section 6 concludes the paper.

## 3. Methodology

In this section, we first introduce the patch representation schema for generating 2D spatio-temporal input patches for STIR-Net. Then, we describe how to synthesize the multi-directional spatio-temporal image restoration network by joint super-resolution and denoising at various cross-sections.

### 3.1. Patch Representation

Three types of patches serve as inputs in this work, consisting of the following: patches for image SR tasks, for denoising tasks, and for conjoint SR and denoising tasks. All the 2D LR patches are generated from the 3D CTP volumes. We use *X* × *Y* × *T* to indicate the three dimensions of the volume, where *X* and *Y* are spatial dimensions and *T* is the temporal dimension. We extract 2D patches along the *X* × *Y* direction as well as along one of the spatial directions with temporal *T* dimension: *X* × *T* and *Y* × *T*. We create 2D LR patches by down-sampling the cross-sectional images in the spatial direction, temporal direction, or both spatial and temporal directions. For instance, using *X* × *T* and *Y* × *T* cross-sections, we remove every other pixel along the T direction to simulate scanning intervals which are two times longer. This corresponds to two times less X-ray radiation exposure in the resulting images. For the denoising task, we simulate the low tube current images by adding spectrum Gaussian noise on the entire CTP volume, with more details in section 4.3. The 2D patches for denoising are generated based on the noisy volumes along the *X* × *T*, *Y* × *T*, and *X* × *Y* cross-sections. For joint SR and denoising tasks, we apply the same scaling strategies that we use to create LR patches, but we apply them on top of noisy volumes. After feeding these LR and/or noisy patches with their labels (the patches extracted from the standard dose) into convolution layers for learning the spatio-temporal details, HR and/or de-noised outputs will be generated in the testing stage based on the captured features.

### 3.2. STIR-Net: Spatial-Temporal Image Restoration Net

Our proposed STIR-Net is a CNN-based end-to-end spatial-temporal architecture for image restoration. To begin, we describe the fundamental SRDN structure—super-resolution denoising networks for cross-section images. Then, we explain in detail the composition of STIR-Net.

#### 3.2.1. SRDN: Super-Resolution Denoising Structure

The usage of kernel combination strategy in GoogLeNet (56) shows that a creative structuring of layers can lead to improved performance and computationally efficiency. Inception modules place various sizes of kernels in parallel. This can extract fine-gain details in volume, while the broader kernel can cover a large receptive field of the input. Extracting diverse information can help with the prediction in classification tasks; however, image denoising poises different challenges.

**Figure 1:**
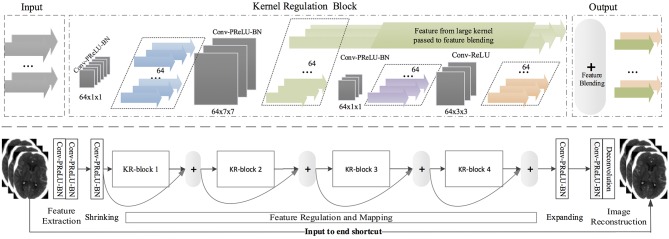
**(Top)** A kernel regulation block (KR-block) with a massive of convolution computations (128 × 7 × 7) comprises two 1 × 1 convolution components for computation reduction and one 3 × 3 convolution module for regularizing the features extracted by the preceding large size kernels. The number of dark-gray blocks indicates the quantity of kernels in the current convolutional layers, and the size of dark-gray blocks represents the size of kernels and the density of convolution. The color arrows represent the quantity of feature-map outputs. **(Bottom)** SRDN is consisted of feature extraction, shrinking, regulation and mapping, expanding, and image reconstruction. Four KR-blocks are embedded in the proposed SRDN.

SRDN is an end-to-end structure that learns from pair-wise LR/noisy patches with their original clean images and outputs high-quality CT images based on low-quality input images while testing. The structure of SRDN is shown in Figure 1. The main functional part of SRDN is built by stacking four modularized Kernel Regulation Blocks (KR-Block). KR-Blocks are inspired by GoogLeNet (56), which has a combination of kernels of varying sizes. Specifically, each block comprises of two 1 × 1 convolutional layers, one 7 × 7 convolutional layer, and one 3 × 3 convolutional layer for regulating the features extracted by the 7 × 7 convolutional layer. The combination of large and small filters is to balance extraction of subtle and edge features. Moreover, each block is embedded with a skip-connection, which allows reference to the feature mapping from previous layers and boosts the network performance.

- **Serial connections**. Image classification needs to summarize diverse information to a linear classifier. On the contrary, image denoising needs to find the most prominent features for a progressive transformation. Therefore, we adopt three kernel sizes (e.g., 1 × 1, 3 × 3, and 7 × 7) in the KR-block module. Kernels of each size are placed in series to allow the small kernels to regulate the features extracted by the large one.
- **Small behind large**. Large kernels (e.g., 7 × 7) can extract certain features by observing a local region with more statistical pixel information. The small kernels (e.g., 3 × 3) are primarily used for exploiting deeper prior information from the underlying feature-maps obtained by large preceding kernels. The subtle textures are especially highlighted during this regularization procedure. Large kernels excel in noise removal but may also smooth the whole image irrespective of its edges or details. Small kernels can preserve subtle textures, but noise pixels may detract from the information attained. Therefore, placing a small kernel behind the large one is a straightforward strategy to enhance the denoiser regularization.
- **Feature blending**. The features extracted by large kernels contain both actual pixel values and noise, whereas the small kernel can capture real pixels while simultaneously ignoring much of the noise. At the end of a KR-block, features captured by small kernels are blended with the features extracted from large kernels. To allow the locally highlighted features to be shared across neighboring KR-blocks, feature-blending is processed by pixel-wise summation (see Figure 1-top) rather than concatenation (e.g., in GoogLeNet). This helps with finding the most prominent features for a forward transformation. Eventually, the output of a KR-block contains more accurate pixel information with less noise.
- **1 × 1** **convolution**. The special usage of 1 × 1 convolution in KR-block is for two purposes: first, it reduces the dimensions inside KR-block modules, such as the first 1 × 1 convolution layer; second, it adds more non-linearity by having PReLU immediately after every 1 × 1 convolution and suffers less from over-fitting due to smaller kernel size.

#### 3.2.2. SRDN Architecture

Convolutional networks learn a mapping function between a corrupted image input and a corresponding noise-free image. The network contains L convolution layers (Conv), each of which implements a feature extraction procedure. To ensure our network has rich feature representations, we use a considerable amount of large filters in the first two convolutional layers (57) to extract diverse and representative features for feature mapping and spatial transformation. We define densely convolutional features extracted from the *l*th layer as

(1) $$\begin{array}{r} x_{l}=Conv{\left(y_{l},f_{l},n_{l},c_{l}\right)}_{f\geq 7\times 7,n\geq 128} \end{array}$$

where *l* = 1…*L* indexes the layer, *y*_*l*_, *f*_*l*_, *n*_*l*_, and *c*_*l*_ represent the *l*'s input, the filter size, filter number, and channel number, respectively. *x*_*l*_ are the feature maps extracted from *y*_*l*_ by *Conv*(·), which denotes convolution. As the top and bottom layers have different functional attentions (57), the network can be decomposed into three parts (the bottom part is shown in Figure 1): feature extraction, feature regulation and mapping, and image reconstruction. In the proposed SRDN, the first two layers have the same volume: (*f*_*l*_, *n*_*l*_, *c*_*l*_) = (7, 128, 1).

Several KR-blocks are cascaded to perform feature regulation, mapping, and transformation. Also, residual learning is performed here by skip-connection, which connects the outputs of two adjacent KR-blocks. The use of skip connection between KR-blocks leads to faster and more stable training. The purpose of using a shortcut between the input and the end of the network is to incorporate more information from the original input into image reconstruction. This strategy helps relax the network interference difficulty because input data contains much real pixel information that can be taken as a prior. To make SRDN more compact, we introduce two 1 × 1 composite units, referred as “Shrinking” and “Expanding,” shown in Figure 1. After densely convolutional feature-extraction layers, we reduce the number of feature maps by “Shrinking.” After feature regulation and mapping, we expand feature maps such that there are sufficient various features that can be provided for image reconstruction. The convolutional layer before the last layer has the volume: (*f*_*l*_, *n*_*l*_, *c*_*l*_) = (3, 128, 1). We utilize a deconvolutional layer with the volume: (*f*_*l*_, *n*_*l*_, *c*_*l*_) = (3, 1, 1) as our last layer.

#### 3.2.3. STIR-Net Structure

The combination of the various features extracted from multi-directional data enhances the network's capability for inference and generality. Since multi-directional inputs provide different perspectives of the 3D volume data, they cannot merely be regarded as feeding more training data into multi-networks. Instead, they complement each other nicely to encode the sparse features through the network.

**Figure 2:**
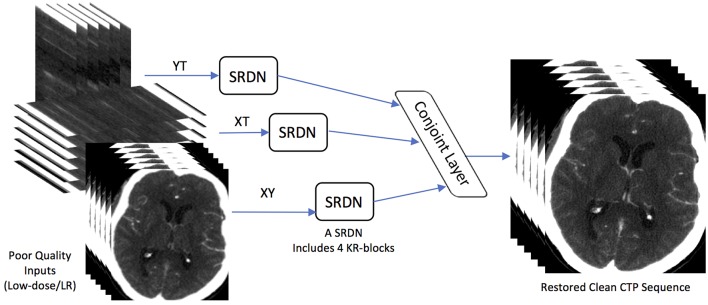
STIR-Net Architecture. STIR-Net takes low-dose inputs from three cross-sections: XY, XT, and YT. Each cross-section go through one SRDN, and the outputs of SRDNs meet in a conjoint layer, which calculates the mean of the three output volumes form SRDNs to provide the final results of STIR-Net.

Dense convolutions and kernel regulation strategy ensure diverse features from multi-directional brain CT images, which can be encoded as network representations. In this paper, we adopt three SRDNs to cope with three directional extracted data respectively: *Y* × *T*, *X* × *T*, and *X* × *Y* to form our STIR-Net. The structure of STIR-Net is shown in Figure 2. During training, the input and output layers are matched with pair-wise noisy and label patches. The label here refers to the patches extracted from the original high radiation dose CTP volume (*X* × *Y* × *T*). Each SRDN contains 4 KR-blocks that can fully encode the features from each directional data without overfitting. For the testing stage, the outputs of the three SRDN nets assemble into a conjoint learning layer. This layer blends various features from all SRDN nets together to be one spatio-temporal volume by calculating the mean of the three outputs.

## 4. Platform and Data Acquisition

### 4.1. Computational Platform

We use the deep learning framework Caffe (58) for constructing the proposed STIR-Net. All experiments are conducted by a GPU workstation that contains four NVIDIA PASCAL xp GPUs. For data preprocessing and post analysis, we use MATLAB (Version R2016b) as it is an efficient programming language for matrix-based image processing.

### 4.2. Datasets

We evaluate the proposed method on 23 stroke patients' CTP sequences. All CTP sequences are scanned using the same acute stroke protocol for patients from August 2007 to June 2010 using GE Lightspeed or Pro-16 scanners (General Electric Medical Systems, Milwaukee, WI). The scanners are in cine 4i scanning mode and perform 45 s acquisitions at one rotation per second using 80 kVp and 190 mAs. Approximately 45 mL of non-ionic iodinated contrast was administered intravenously at 5 mL/s using a power injector with a 5 s delay. The thickness of the brain region at the z-axis is 20 mm for each sequence, and each sequence has four slices along the z-axis where each slice is 5 mm thick (cross-plane resolution). The brain region has 0.43 spatial resolution (in-plane resolution) on the xy-plane. The slices within one CTP sequence are intensity normalized and co-registered over time. The entire volume size of one patient is 512 × 512 × 4 × 119, where 512 is the height and width of each CT slice, 4 is the number of slices on the z-axis, and 119 is the number of frames in the CTP sequence. In this paper, we only select one slice along the z-axis, thus the size of resulting the CTP volume is 512 × 512 × 119, denoted as *X* × *Y* × *T*.

We randomly split the patients into three groups: 12 patients for training, four patients for validation, and seven patients for testing. As each patient has 119 slices, the training, validation, and testing set resulted in 1,428, 476, and 833 images in XY cross-section (the spatial direction), respectfully. We only maintain brain regions in the images for the other two cross-sections, XT and YT, or about 300 pixels for the X and the Y directions. Therefore for these cross-sections, we estimate that we have 3,600 images for training, 1,200 for validation, and 2,100 for testing. We use the patch-based method in this paper, so the images are further cropped into patches of size 41 × 41 with a stride of 21. This resulted in 822,528 and 274,176 patches in XY cross-section, 75,600 patches in XT cross-section, and 25,200 patches in YT cross-section, respectively for training and validation.

### 4.3. Low Radiation Dose Simulation and Data Preprocessing

To simulate low radiation dose CTP images, we address three generation approaches: reducing the tube current, shortening X-ray radiation exposure time, and lowering spatial resolution. We detail each criterion as below.

- **Low Tube Current**. We followed the same steps described in Britten et al. (59) to simulate the low-dose CT images by adding spatially correlated statistical noise (spectrum Gaussian noise). The generated noise is directly added on the original high-dose images, where the high-dose volumes are scanned at tube current *I*_0_ = 190*mAs*. Based on Britten et al. (59), the noise model is built on the inverse relationship between the tube current *I* and the noise standard deviation σ in CT images. The noise level σ (the standard deviation of Gaussian noise that we want to add to the original images) is adjusted based on tube current *I* that we want to simulate according to equation
  (2) $$\begin{array}{r} \sigma =K\times \sqrt{\frac{1}{I}-\frac{1}{I_{0}}} \end{array}$$
  where $K=103.09mA^{\frac{1}{2}}$ is computed based on phantom studies. We simulate four levels of noisy images in this paper at different tube currents: 20, 40, 60, and 80 mAs.
- **Low Temporal Sampling Rate**. To reduce the temporal sampling rate for shorter X-ray radiation exposure time, we simulate longer scanning intervals by removing frames between specific time intervals. For example, we remove every other frame from the CTP volume to generate the down-sampled volume that is two times shorter on the temporal dimension than the original length. In this way, we skip frames with two scales *S*_*i*_: two times shorter *S*_2_ and three times *S*_3_ shorter than the original time. We also keep the original length *S*_1_ for comparison. For all down-sampled volumes, we scale them back to the original size via bicubic interpolation for deep learning experiments.
- **Low Spatial Sampling Rate**. We lower the CT spatial sampling rate to mimic the low spatial resolution images that are produced by a limited amount of beams and receptors. For instance, we create the down-sampled images by skipping every other pixel (scaling rate of two) along the X and Y directions in the original high radiation dose images respectively (so-called grid-wise). We simulate the LR images by skipping pixels grid-wise with two scales *S*_*i*_: two times down-sampled *S*_2_, and three times down-sampled *S*_3_. We set *S*_1_ as no down-sampling for comparison. Then, we interpolate the down-sampled images by the bicubic method to scale them back to the original image size.

Based on different patch representations that are described in section 3.1, we preprocess the data subsequently. We have three combinations of directional cross-sections XY, XT, and YT for STIR-Net. For each individual denoising and super-resolution case, we add Gaussian noise to the high-dose images and apply spatio/temporal down-sampling, respectively. For the combination of super-resolution and de-noising, we add the noise first and then apply spatial/temporal down-sampling depending on different scaling factors.

## 5. Experiments and Results

The experiments of this work are carried out in three steps: image super-resolution, image denoising, and image super-resolution with denoising. In the first two steps, we want to show that the proposed STIR-Net is capable of different image restoration tasks independently. Further, in the third step, we want to demonstrate that our STIR-Net can tackle super-resolution and denoising simultaneously. We train the STIR-Net structure from scratch using low-quality images from different cross-sections, then we test each of the cross-sections as spatial-only, temporal-only, and spatial and temporal combined. The performance is computed based on the average result form seven patients' 119 slices. As cross-sections (XT and YT) are trained and tested in a 2D circumstance that combined temporal dimension with one spatial dimension, we concatenate the resulted 2D images into 3D volumes and recalculate the performance based on XY direction.

### 5.1. Evaluation Metrics

The experiment performance is evaluated based on two evaluation metrics: structural similarity (SSIM) index and PSNR. SSIM is used for measuring the similarity between two images based on the computation of luminance term *l*(*x, y*), the contrast term *c*(*x, y*), and the structural term *s*(*x, y*), where *x* and *y* are two images. We calculate SSIM based on the following equations

(3) $$\begin{array}{rcl} SSIM\left(x,y\right) & = & \left[l\left(x,y\right)\cdot c\left(x,y\right)\cdot s\left(x,y\right)\right] \end{array}$$

(4) $$\begin{array}{rcl} l\left(x,y\right)=\frac{2\mu_{x}\mu_{y}+c_{1}}{\mu_{x}^{2}+\mu_{y}^{2}+c_{1}},c\left(x,y\right) & = & \frac{2\sigma_{x}\sigma_{y}+c_{2}}{\sigma_{x}^{2}+\sigma_{y}^{2}+c_{2}},\\ s\left(x,y\right) & = & \frac{2\sigma_{x}y+c_{3}}{\sigma_{x}^{2}+\sigma_{y}^{2}+c_{3}} \end{array}$$

where μ_*x*_, μ_*y*_, σ_*x*_, σ_*y*_, σ_*xy*_ are the local means, standard deviations, and cross-covariance for images *x* and *y*. The value of *c*_1_, *c*_2_, and *c*_3_ are set as 6.5025, 58.5225, and 29.26125, where the values are calculated based on the dynamic range *L* of the pixel-values (here is 255) in function $c_{1}={\left(0.01*L\right)}^{2}$, $c_{2}={\left(0.03*L\right)}^{2}$, and *c*_3_ = *c*_2_/2. PSNR defines the ratio between the maximum intensity value in the ground truth image *I*_*max*_ and the power of corrupting noise σ (root mean square error between the ground truth and enhanced image) that affects representation fidelity.

(5) $$\begin{array}{r} PSNR=20\log_{10}\frac{I_{max}}{\sigma } \end{array}$$

### 5.2. Image Super-Resolution

The first experiment is image super-resolution, which is independently conducted on three cross-sections (*Y* × *T*, *X* × *T*, and *X* × *Y*) at two sampling rates (*S*_2_: down-sampling to 1/2, *S*_3_: down-sampling to 1/3). We want to evaluate whether the proposed STIR-Net is capable of achieving a stable performance in different cross-sections at different levels of scaling. For the XY cross-section, we down-sample along the spatial directions to create low-resolution images. For the XT and YT cross-sections, we down-sample on the temporal direction only to simulate scanning in a shorter X-ray radiation exposure time. The experimental results of STIR-Net are shown in Table 1. We calculate SSIM and PSNR values for LR inputs, SR outputs, and the improvements of SR from LR. The greatest improvements for both SSIM and PSNR are in the XY direction, while the XT and YT directions have achieved similar improvements. When the sampling rate is high, the improvements compared to the lower sampling rate are higher in almost all cross-sections. The improvements of SSIM and PSNR are highly stable and follow the same trend in different conditions. A one-tailed paired *t*-test was conducted to compare the performance improvements of PSNR and SSIM values. There was a significant difference in the scores for PSNR (Mean = 37.623, SD = 10.955) and SSIM (Mean = 0.950, SD = 0.001) before and after using the proposed method; where *p* = 0.0003 for PSNR and *p* = 0.0004 for SSIM show that the improvements are significant as *p* < 0.05. These results suggest that PSNR and SSIM do improve significantly after applying our model in this experiment. This experiment indicates that STIR-Net has the potential to address low spatial and temporal resolution in CTP image volumes.

**Table 1:** Average SSIM and PSNR (dB) performance of seven patients' 833 CTP slices between different sampling scales for STIR-Net image super-resolution at different spatio-temporal cross-sections.

**Direction**	**Scale**	**SSIM**			**PSNR**		
		**LR**	**STIR-Net**	**Improvement**	**LR**	**STIR-Net**	**Improvement**
XY	*S*_2_	0.954	0.987	0.033	32.348	43.195	10.846
	*S*_3_	0.855	0.929	0.074	26.958	35.317	8.359
YT	*S*_2_	0.939	0.971	0.032	33.354	38.961	5.607
	*S*_3_	0.887	0.929	0.042	29.240	35.167	5.926
XT	*S*_2_	0.929	0.967	0.038	32.965	38.580	5.615
	*S*_3_	0.866	0.915	0.048	28.831	34.513	5.681

### 5.3. Image Denoising

In this experiment, we explore different levels of low tube current for training STIR-Net. We added the spectrum Gaussian noise to simulate four low tube currents: 20, 40, 60, and 80 mAs, which are 11, 21, 32, and 42% of the original 190 mAs tube current. We train the proposed STIR-Net by mixing together the different tube currents - it is more difficult to restore high-dose images at lower tube current, as shown in Table 2. This table shows that the SSIM and PSNR performances for the XY direction when STIR-Net is trained and tested with mixed levels of tube currents, which are at a fixed spatial/temporal sampling rate of *S*_2_. The improvement of SSIM increases as tube currents decrease, while the improvement of PSNR remains in a similar range. We show that STIR-Net is a general solution for different tube currents, as the PSNR improvements for different test cases are all higher than 5 dB. In this experiment, we demonstrate that STIR-Net can tackle denoising problems as well, even for mixed noise levels. The improvements are very stable for different tube current levels.

**Table 2:** Average SSIM and PSNR (dB) performance of seven patients' 833 slices for XY direction when STIR-Net is trained and tested with mix levels of tube currents, where at a fixed spatial/temporal sampling scale *S*_*2*_.

**mAs**	**SSIM**			**PSNR**		
	**LR**	**STIR-Net**	**Improvement**	**LR**	**STIR-Net**	**Improvement**
20	0.778	0.859	0.080	25.227	30.445	5.217
40	0.830	0.896	0.065	26.663	32.349	5.686
60	0.860	0.909	0.049	27.323	33.062	5.739
80	0.879	0.915	0.036	27.702	33.409	5.706

*We show that STIR-Net is a general solution for different tube currents as the PSNR improvements for different test cases are all higher than 5 dB. mAs is the unit for tube current-time product*.

### 5.4. Spatial-Temporal Super-Resolution and Denoising

In addition to the encouraging individual experiment results for image super-resolution and denoising, the experiment results in both spatial and temporal super-resolution with denoising have also achieved great enhancements. We evaluate the resulted images based on two aspects in this section: the analysis on the resulted CTP sequence and the analysis on the generated perfusion maps.

#### 5.4.1. CTP Sequence Analysis

Table 3 shows the PSNR comparison of the resulted CTP sequence among Multi-Scale Expected Patch Log Likelihood (MS-EPLL) (60) method, our previously proposed method STAR, and the current method STIR-Net. The test results are displayed as an average value over seven test patients' 833 slices output. The STAR and STIR-Net methods both contain three scenarios: spatial SR only, temporal SR only, and joint spatial and temporal SR. In both methods, the temporal SR includes two cross-sections (the XT and YT directions).

**Table 3:** Average PSNR comparison of seven patients' 833 CTP slices for different conditions.

**mAs**	**Scale**	**LR**	**MS-EPLL**	**STAR**			**STIR-Net**		
			**Spatial**	**Spatial**	**Temporal**	**Conjoint**	**Spatial**	**Temporal**	**Conjoint**
20	S_1_	23.438	28.566	31.734	32.550	**33.273**	31.856	32.456	33.189
	S_2_	23.032	26.022	28.705	31.006	**31.017**	28.847	30.774	30.919
	S_3_	21.993	22.517	26.636	29.990	29.682	26.861	**30.405**	30.096
40	S_1_	26.764	31.176	33.763	34.511	**35.452**	33.848	34.685	35.402
	S_2_	24.698	27.357	29.899	31.841	32.214	30.092	32.471	**32.659**
	S_3_	23.246	23.576	27.289	31.020	30.609	27.555	**31.272**	30.909
60	S_1_	29.079	33.030	35.336	36.145	**37.254**	35.415	36.064	37.195
	S_2_	25.498	28.161	30.705	33.269	33.489	30.956	33.544	**33.913**
	S_3_	23.801	24.689	27.694	31.673	31.207	27.785	**31.867**	31.450
80	S_1_	31.025	34.607	36.769	37.664	**38.967**	36.854	36.724	38.469
	S_2_	25.971	28.608	31.505	34.123	34.464	31.719	34.324	**34.728**
	S_3_	24.116	24.658	28.027	32.268	31.706	27.688	**32.438**	31.687
Avg		25.222	27.748^*^	30.672${}_{⋆}^{*}$	33.005${}_{⋆}^{*}$	33.278${}_{⋆}^{*}$	30.790${}_{⋆}^{*}$	33.086${}_{⋆}^{*}$	**33.385**${}_{⋆}^{*}$

*In this table, three methods are compared: MS-EPLL, STAR, and STIR-Net. The conditions include four types of tube current (20, 40, 60, and 80 mAs) and three kinds of SR scales (S_1_: no down-sampling, S_2_: down-sampling to 1/2, and S_3_: down-sampling to 1/3). LR means the PSNR value for the noise image after down-sampling. S_1_ is image denoising only. The best values are highlighted for different scenarios. The average value is listed at the bottom of the table. The asterisk symbol denotes the result of the current method achieves significant higher PSNR value than the LR images at α = 0.05 when performing the one-tailed paired t-tests, and the star symbol denotes the comparison between MS-EPLL method*.

Table 3 focuses on the comparison of four levels of tube current (20, 40, 60, 80 mAs) and three SR scales (*S*_1_: no down-sampling, *S*_2_: down-sampling to 1/2, *S*_3_: down-sampling to 1/3). The down-sample rates are applied based on different methods: spatial-only models are scaled down on the spatial dimensions, temporal-only models are scaled down on the temporal dimension, and the conjoint models are scaled down on both spatial and temporal dimensions (depending on different cross-sections). In this table, LR refers to the PSNR value for the noise image after down-sampling. We highlighted the best values for different scenarios. From this table, we can see STAR achieves higher PSNR for denoising than STIR-Net, while STIR-Net performs better for mixed noise and down-sampling scenarios. Moreover, both STAR and STIR-Net methods outperform the MS-EPLL method. For all tube currents, the PSNR value follows the trend of better image restoration results at higher tube currents. Similarly, a lower down-sampling rate leads to better reconstruction performance. The conjoint of spatial and temporal directions of STAR gives the best results for all four tube current levels. When the low dose CT images have poor spatial or temporal resolutions, it is usually more difficult to tackle both denoising and SR problems; however, our STIR-Net net is more favorable for these situations. Its conjoint model gives us an average 32% improvement from the LR inputs. The experiment results indicate that most mixed low dose and low-resolution scenarios can achieve the best performances, especially for the temporal directions. This means that for the temporal directions, there is more related information that can be used for reconstructing CT frames that are nearby the down-sampled slices. The average performance improvement for STIR-Net net is about 8.08 dB from the LR inputs and around 4 dB compared to the MS-EPLL method. We perform one-tailed paired *t*-tests in Table 3 to compare PSNR values at different mAs and super-resolution scales using *alpha* = 0.05. All three types of STIR-Net perform significantly better than LR and MS-EPLL, especially the conjoint model achieves the best performance among all methods.

#### 5.4.2. Perfusion Maps Analysis

We compare the perfusion maps (CBF and CBV) based on which physicians make the clinical decision, as the perfusion maps can show the hemodynamic changes of blood flow. Therefore, achieving higher accuracy in restoration in perfusion maps is critical for clinical diagnosis.

**Figure 3:**
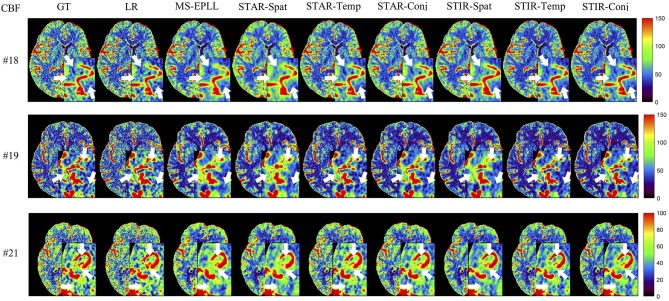
Visual comparison of CBF for three test patients: #18, #19, and #21, when reducing the tube current to 40 mAs with a down-sample ratio of two (two times low spatial and two time low temporal resolutions). The notation for each column is: GT, Ground truth image; LR, Low-Resolution input; MS-EPLL, MS-EPLL restoration result; STAR-Spat, STAR reconstruction result (spatial only); STAR-Temp, STAR reconstruction result (temporal only); STAR-Conj, STAR reconstruction result (spatial + temporal); STIR-Spat, STIR-Net reconstruction result (spatial only); STIR-Temp, STIR-Net reconstruction result (temporal only); STIR-Conj, STIR-Net reconstruction result (spatial + temporal). All figures are displayed by using the same colormap and the color range for each patient is shown in the colorbar on the rightmost of each row. We use white arrows to compare the details in the region of interests.

**Figure 4:**
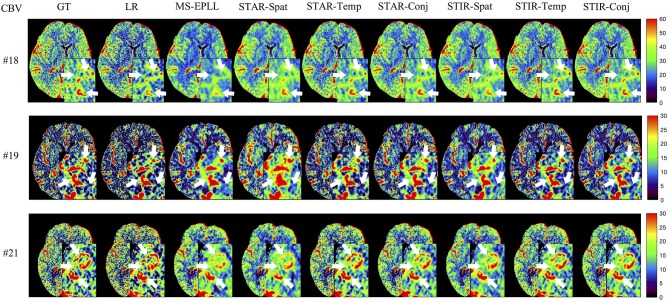
Visual comparison of CBV for three test patients: #18, #19, and #21, when reducing the tube current to 40 mAs with a down-sample ratio of two (two times low spatial and two time low temporal resolutions). The notation for each column is: GT, Ground truth image; LR, Low-Resolution input; MS-EPLL, MS-EPLL restoration result; STAR-Spat, STAR reconstruction result (spatial only); STAR-Temp, STAR reconstruction result (temporal only); STAR-Conj, STAR reconstruction result (spatial + temporal); STIR-Spat, STIR-Net reconstruction result (spatial only); STIR-Temp, STIR-Net reconstruction result (temporal only); STIR-Conj, STIR-Net reconstruction result (spatial + temporal). All figures are displayed by using the same colormap and the color range for each patient is shown in the colorbar on the rightmost of each row. We use white arrows to compare the details in the region of interests.

**Figure 5:**
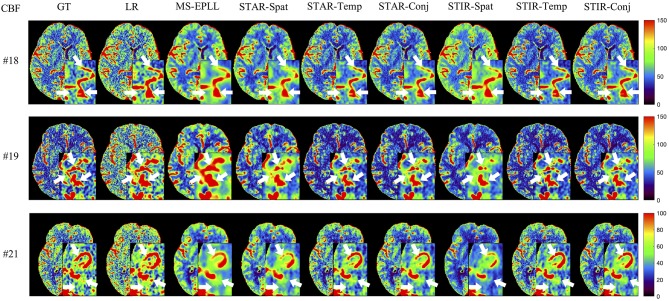
Visual comparison of CBF for three test patients: #18, #19, and #21, when reducing the tube current to 40 mAs with a down-sample ratio of three (three times low spatial and two time low temporal resolutions). The notation for each column is: GT, Ground truth image; LR, Low-Resolution input; MS-EPLL, MS-EPLL restoration result; STAR-Spat, STAR reconstruction result (spatial only); STAR-Temp, STAR reconstruction result (temporal only); STAR-Conj, STAR reconstruction result (spatial + temporal); STIR-Spat, STIR-Net reconstruction result (spatial only); STIR-Temp, STIR-Net reconstruction result (temporal only); STIR-Conj, STIR-Net reconstruction result (spatial + temporal). All figures are displayed by using the same colormap and the color range for each patient is shown in the colorbar on the rightmost of each row. We use white arrows to compare the details in the region of interests.

**Figure 6:**
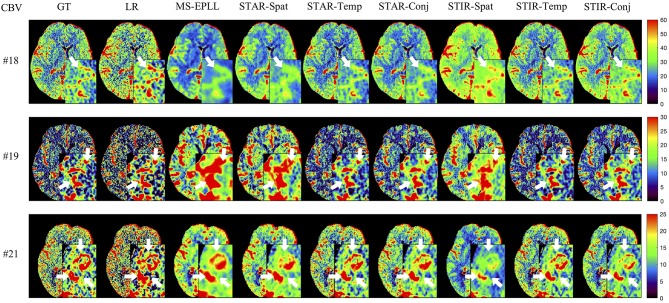
Visual comparison of CBV for three test patients: #18, #19, and #21, when reducing the tube current to 40 mAs with a down-sample ratio of three (three times low spatial and two time low temporal resolutions). The notation for each column is: GT, Ground truth image; LR, Low-Resolution input; MS-EPLL, MS-EPLL restoration result; STAR-Spat, STAR reconstruction result (spatial only); STAR-Temp, STAR reconstruction result (temporal only); STAR-Conj, STAR reconstruction result (spatial + temporal); STIR-Spat, STIR-Net reconstruction result (spatial only); STIR-Temp, STIR-Net reconstruction result (temporal only); STIR-Conj, STIR-Net reconstruction result (spatial + temporal). All figures are displayed by using the same colormap and the color range for each patient is shown in the colorbar on the rightmost of each row. We use white arrows to compare the details in the region of interests.

**Visual Comparison:** The visual comparisons of the generated perfusion maps (CBF and CBV) are presented in Figures 3–6 for patient # 18, # 19, and # 21 in the case of scale level *S*_2_ and *S*_3_ with 40 mAs. We enlarge the region of interest for each image to check the details, and we highlight the details by using white arrows. From these figures, the edges in the LR images are distorted compared to the original images, and MS-EPLL restores the detail information incorrectly. The resulting images of the STIR-Net models are much closer to the ground truth images compare to MS-EPLL and STAR. The boundaries and details of the features in STIR-Net results are well-preserved, and the figures are less blurry than other methods. In sum, the proposed STIR-Net gives us much accurate perfusion maps compare to MS-EPLL and STAR methods as it restores the edge information much closer to the ground truth images.

**Quantitative Comparison:** We calculate the CBF and CBV values based on the CTP sequences resulted from different methods. Then, we use PSNR and SSIM as evaluation metrics. As the proposed method STIR-Net is designed for CTP image super-resolution and denoising simultaneously, we show the results of 40 mAs at the down-sample scale of *S*_2_ and *S*_3_. Tables 4, 5 provide the PSNR and SSIM comparisons of CBF and CBV maps in the case of scale level *S*_2_ with 40 mAs and Tables 6, 7 are for scale level *S*_3_. In general, STIR-Net models achieve the best performance, and the temporal model is usually the top performer.

**Table 4:** PSNR and SSIM value comparison of seven patients' CBF maps calculated at scale *S*_*2*_ with tube current 40 mAs.

**CBF**	**Patient**	**LR**	**MS-EPLL**	**STAR**			**STIR**		
			**Spatial**	**Spatial**	**Temporal**	**Conjoint**	**Spatial**	**Temporal**	**Conjoint**
PSNR	#18	24.57	25.74	27.83	30.54	**31.30**	28.06	29.07	29.79
	#19	23.87	25.18	26.31	**28.91**	28.81	26.25	27.83	27.84
	#20	23.07	26.09	29.30	30.94	31.63	24.16	32.02	**32.31**
	#21	25.34	26.64	28.02	**31.70**	31.59	27.86	31.26	30.70
	#22	23.25	26.13	27.90	30.46	30.86	28.26	30.24	**31.14**
	#23	23.84	25.16	27.37	27.26	**28.99**	27.87	26.24	27.11
	#24	18.23	21.59	23.55	**26.62**	26.61	24.68	25.89	26.30
	Avg	23.15	25.21^*^	27.18${}_{⋆}^{*}$	29.49${}_{⋆}^{*}$	**29.97**${}_{⋆}^{*}$	26.73${}_{⋆}^{*}$	28.94${}_{⋆}^{*}$	29.31${}_{⋆}^{*}$
	Var	5.28	2.80	3.34	3.77	3.59	2.95	5.74	5.09
SSIM	#18	0.84	0.85	0.88	0.93	0.93	0.88	**0.94**	0.93
	#19	0.77	0.80	0.82	0.89	0.89	0.81	**0.90**	**0.90**
	#20	0.75	0.84	0.86	0.91	0.92	0.78	**0.94**	0.93
	#21	0.78	0.82	0.83	0.90	0.91	0.83	**0.92**	0.91
	#22	0.78	0.83	0.86	0.92	0.92	0.86	**0.93**	**0.93**
	#23	0.82	0.84	0.86	0.89	**0.92**	0.87	0.89	0.89
	#24	0.72	0.77	0.80	0.88	0.89	0.82	**0.90**	0.89
	Avg	0.78	0.82^*^	0.84${}_{⋆}^{*}$	0.90${}_{⋆}^{*}$	0.91${}_{⋆}^{*}$	0.83^*^	**0.92**${}_{⋆}^{*}$	0.91${}_{⋆}^{*}$
	Var	0.0016	0.0008	0.0008	0.0003	0.0002	0.0014	0.0004	0.0004

*In this table, three methods are compared: MS-EPLL, STAR, and STIR-Net. LR means the PSNR value for the noise image after down-sampling. The best values are highlighted for different patients. The average value and the variance are listed at the bottom of the table. The asterisk symbol denotes the result of the current method achieves significant higher PSNR value than the LR images at α = 0.05 when performing the one-tailed paired t-tests, and the star symbol denotes the result is significantly higher than MS-EPLL method*.

**Table 5:** PSNR and SSIM value comparison of seven patients' CBV maps calculated at scale *S*_*2*_ with tube current 40 mAs.

**CBV**	**Patient**	**LR**	**MS-EPLL**	**STAR**			**STIR**		
			**Spatial**	**Spatial**	**Temporal**	**Conjoint**	**Spatial**	**Temporal**	**Conjoint**
PSNR	#18	28.00	28.62	31.78	34.24	**35.22**	31.68	34.66	34.17
	#19	32.32	34.05	33.79	37.52	38.11	35.15	**38.75**	38.63
	#20	30.62	32.83	37.53	38.30	39.69	34.80	**40.15**	39.77
	#21	32.20	33.38	34.95	37.67	37.98	35.09	38.07	**38.27**
	#22	31.60	32.76	32.70	37.80	37.57	33.15	**38.94**	38.50
	#23	30.55	31.51	32.03	35.87	36.46	34.76	35.37	**35.97**
	#24	26.42	29.37	31.37	32.53	33.28	30.74	**35.37**	33.55
	Avg	30.22	31.76^*^	33.44${}_{⋆}^{*}$	36.26${}_{⋆}^{*}$	36.89${}_{⋆}^{*}$	33.60${}_{⋆}^{*}$	**37.08**${}_{⋆}^{*}$	36.97${}_{⋆}^{*}$
	Var	4.87	4.16	4.77	4.67	4.47	3.18	6.11	5.82
SSIM	#18	0.86	0.87	0.90	0.94	0.94	0.90	**0.95**	0.94
	#19	0.84	0.87	0.87	0.92	0.93	0.88	**0.94**	**0.94**
	#20	0.89	0.92	0.93	0.96	0.96	0.93	**0.97**	**0.97**
	#21	0.84	0.88	0.89	0.92	0.93	0.89	**0.94**	0.93
	#22	0.85	0.88	0.89	0.93	0.94	0.89	**0.95**	**0.95**
	#23	0.85	0.88	0.89	0.92	**0.93**	0.90	**0.93**	**0.93**
	#24	0.77	0.82	0.84	0.88	0.89	0.84	**0.90**	**0.90**
	Avg	0.84	0.88^*^	0.89${}_{⋆}^{*}$	0.93${}_{⋆}^{*}$	0.93${}_{⋆}^{*}$	0.89${}_{⋆}^{*}$	**0.94**${}_{⋆}^{*}$	**0.94**${}_{⋆}^{*}$
	Var	0.0015	0.0008	0.0008	0.0005	0.0004	0.0007	0.0004	0.0004

*In this table, three methods are compared: MS-EPLL, STAR, and STIR-Net. LR means the PSNR value for the noise image after down-sampling. The best values are highlighted for different patients. The average value and the variance are listed at the bottom of the table. The asterisk symbol denotes the result of the current method achieves significant higher PSNR value than the LR images at α = 0.05 when performing the one-tailed paired t-tests, and the star symbol denotes the result is significantly higher than MS-EPLL method*.

**Table 6:** PSNR and SSIM comparison of seven patients' CBF maps calculated at scale *S*_*3*_ with tube current 40 mAs.

**CBF**	**Patient**	**LR**	**MS-EPLL**	**STAR**			**STIR**		
			**Spatial**	**Spatial**	**Temporal**	**Conjoint**	**Spatial**	**Temporal**	**Conjoint**
PSNR	#18	21.68	23.89	24.92	27.88	27.30	24.64	**28.09**	27.92
	#19	19.95	20.55	23.39	26.51	26.60	23.71	**27.09**	26.59
	#20	22.41	26.23	25.57	**29.84**	29.62	26.14	29.51	29.04
	#21	23.35	24.85	25.11	28.10	27.54	23.18	**29.36**	27.77
	#22	21.97	23.95	24.23	23.97	28.04	24.83	**28.54**	28.29
	#23	22.50	24.03	23.95	26.05	**26.35**	23.85	24.85	25.84
	#24	17.21	21.45	20.66	**24.53**	23.91	20.91	24.04	23.49
	Avg	21.29	23.56^*^	23.97^*^	26.70${}_{⋆}^{*}$	27.05${}_{⋆}^{*}$	23.89^*^	**27.35**${}_{⋆}^{*}$	26.99${}_{⋆}^{*}$
	Var	4.30	3.77	2.67	4.31	3.07	2.66	4.66	3.51
SSIM	#18	0.74	0.75	0.77	**0.88**	0.87	0.79	**0.88**	**0.88**
	#19	0.64	0.69	0.71	**0.83**	**0.83**	0.71	**0.83**	0.82
	#20	0.70	0.76	0.73	**0.87**	**0.87**	0.77	**0.87**	0.86
	#21	0.68	0.70	0.73	0.82	0.82	0.70	**0.85**	0.84
	#22	0.69	0.75	0.76	0.83	**0.87**	0.77	**0.87**	**0.87**
	#23	0.73	0.74	0.76	**0.85**	**0.85**	0.76	0.84	**0.85**
	#24	0.63	0.68	0.69	**0.82**	**0.82**	0.69	**0.82**	0.81
	Avg	0.69	0.72^*^	0.74${}_{⋆}^{*}$	**0.85**${}_{⋆}^{*}$	**0.85**${}_{⋆}^{*}$	0.74${}_{⋆}^{*}$	**0.85**${}_{⋆}^{*}$	**0.85**${}_{⋆}^{*}$
	Var	0.0016	0.0009	0.0008	0.0006	0.0007	0.0015	0.0005	0.0006

*In this table, three methods are compared: MS-EPLL, STAR, and STIR-Net. LR means the PSNR value for the noise image after down-sampling. The best values are highlighted for different patients. The average value and the variance are listed at the bottom of the table. The asterisk symbol denotes the result of the current method achieves significant higher PSNR value than the LR images at α = 0.05 when performing the one-tailed paired *t*-tests, and the star symbol denotes the result is significantly higher than MS-EPLL method*.

**Table 7:** PSNR and SSIM comparison of seven patients' CBV maps calculated at scale *S*_*3*_ with tube current 40 mAs.

**CBV**	**Patient**	**LR**	**MS-EPLL**	**STAR**			**STIR**		
			**Spatial**	**Spatial**	**Temporal**	**Conjoint**	**Spatial**	**Temporal**	**Conjoint**
PSNR	#18	24.94	27.53	29.48	32.33	31.44	27.43	**34.13**	31.43
	#19	30.71	26.58	28.50	36.15	35.01	29.86	**36.39**	35.48
	#20	28.40	34.42	31.90	37.98	38.11	34.22	**38.49**	38.17
	#21	30.45	31.87	32.83	35.86	35.52	31.36	**35.97**	34.87
	#22	29.28	30.51	29.96	**36.00**	35.83	28.05	**36.00**	35.77
	#23	28.83	31.31	29.08	**35.08**	33.86	27.33	34.35	34.23
	#24	24.96	27.44	28.68	30.22	30.76	28.53	30.87	**31.01**
	Avg	28.21	29.93	30.03 ^*^	34.80${}_{⋆}^{*}$	34.35${}_{⋆}^{*}$	29.52	**35.16**${}_{⋆}^{*}$	34.42${}_{⋆}^{*}$
	Var	5.60	8.15	2.61	6.93	6.59	6.25	5.67	6.30
SSIM	#18	0.77	0.81	0.83	0.90	0.90	0.83	**0.91**	0.90
	#19	0.77	0.76	0.77	0.89	0.89	0.78	**0.90**	0.89
	#20	0.81	0.89	0.85	**0.94**	**0.94**	0.89	**0.94**	**0.94**
	#21	0.77	0.84	0.85	**0.90**	**0.90**	0.83	**0.90**	0.89
	#22	0.76	0.82	0.82	**0.91**	**0.91**	0.80	**0.91**	**0.91**
	#23	0.76	0.84	0.83	**0.90**	**0.90**	0.82	**0.90**	**0.90**
	#24	0.69	0.76	0.77	0.84	**0.85**	0.77	**0.85**	**0.85**
	Avg	0.76	0.82^*^	0.82^*^	**0.90**${}_{⋆}^{*}$	**0.90**${}_{⋆}^{*}$	0.82^*^	**0.90**${}_{⋆}^{*}$	**0.90**${}_{⋆}^{*}$
	Var	0.0013	0.0022	0.0012	0.0009	0.0008	0.0016	0.0007	0.0008

*In this table, three methods are compared: MS-EPLL, STAR, and STIR-Net. LR means the PSNR value for the noise image after down-sampling. The best values are highlighted for different patients. The average value and the variance are listed at the bottom of the table. The asterisk symbol denotes the result of the current method achieves significant higher PSNR value than the LR images at α = 0.05 when performing the one-tailed paired t-tests, and the star symbol denotes the result is significantly higher than MS-EPLL method*.

We perform one-tailed paired *t*-tests for each table to compare PSNR and SSIM of the restored images with LR images and restored images using MS-EPLL and STAR models. The hypothesis for all *t*-tests is: after using the proposed method, we can achieve significant improvements in PSNR and SSIM values from the images of LR, MS-EPLL method, or STAR models. The results show that our proposed STIR-Net models not only significantly improve the PSNR and SSIM values from the LR images but also achieves significantly higher PSNR and SSIM values than the MS-EPLL method, especially for the temporal models and the conjoint models. For comparison with STAR model, Table 4 shows that at *S*_2_ and 40 mAs, CBF's SSIM values using the STIR-Net temporal model is significantly (*p* = 0.002067) better than the STAR temporal model, similar for CBV (*p* = 0.01554). STIR-Net's conjoint model is also significantly better than the STAR conjoint model (*p* = 0.00994) in terms of SSIM. In Table 7, for the case of *S*_3_ and 40 mAs, similar observations are made. STIR-Net temporal model is significantly (*p* = 0.03521) better than the STAR temporal model and conjoint model in terms of both PSNR and SSIM.

Overall, the test results demonstrate the advantage of our STIR-Net to restore high-quality scans at as low as 11% of absorbed radiation dose of the current imaging protocol, yielding an average of 17% improvement in PSNR and SSIM values for perfusion maps including CBF and CBV compared to LR images and 10% improvements compared to MS-EPLL method. For the comparison of STIR-Net and STAR, we calculate the improvements by averaging out all three models including the spatial model, temporal model, and the conjoint model. Our proposed STIR-Net method achieves an average of 0.2% improvements in PSNR and SSIM values for perfusion maps than STAR models.

## 6. Conclusion

This paper presents a novel deep learning-based multi-directional spatio-temporal framework to recover the low radiation dose CTP images of acute stroke patients by addressing both denoising and super-resolution problems simultaneously. Our proposed framework, called STIR-Net, is an end-to-end image restoration network that is capable of recovering images scanned at low tube current, short X-ray radiation exposure time, and low spatial resolution jointly. We emphasize the characteristic of our proposed STIR-Net in CTP image super-resolution and denoising jointly, which directs prior and data fidelity terms with two insights: First, a well-trained CNN-based denoiser can be regarded as a sequence of filter-based denoisers. Second, each component of a CNN-based denoiser has the capacity of jointly dealing with image denoising and super-resolution problems. By combining the cross-sectional features in the spatio-temporal domain, our STIR-Net achieves to better reconstruction results, especially for mixed low-resolution and noise cases. After inputting low dose and low-resolution patches at different cross-sections of the spatio-temporal data simultaneously, STIR-Net blends the features from both spatial and temporal domains to reconstruct high-quality CT volumes. The experimental results indicate that our framework has the potential to maintain the diagnostic image quality not only for reducing the tube current down to 11% of the commercial standard but also for 1/3 X-ray radiation exposure time and 1/3 spatial resolution. Hence, our approach is an efficient and effective solution for radiation dose reduction in CTP imaging. In the future, we will extend the work into multimodal imaging radiation dose reduction by combining low-dose non-contrast CT, CTA, and CTP images holistically.

## Author Contributions

YX drafted the manuscript, designed the STIR-Net architecture and the experiments, carried out the experiments and analysis. PL designed the SRDN deep learning network structure, drafted the manuscript SRDN section. YL assisted the generation of the perfusion maps and the related analysis. SS, PS, AG, and JI revised the manuscript critically for important intellectual content. RF designed and directed the project.

### Conflict of Interest Statement

The authors declare that the research was conducted in the absence of any commercial or financial relationships that could be construed as a potential conflict of interest.

## References

[1] Yang Q Tong X Schieb L Vaughan A Gillespie C Wiltz JL. Vital signs: recent trends in stroke death rates-United States, 2000-2015. Morb Mortal Wkly Rep. (2017) 66:933–9. 10.15585/mmwr.mm6635e1 28880858 PMC5689041

[2] Hall MJ Levant S DeFrances CJ Hospitalization for stroke in US hospitals, 1989–2009. Diabetes. (2012) 18:23. 22617404

[3] Benjamin EJ Blaha MJ Chiuve SE Cushman M Das SR Deo R. Heart disease and stroke statistics-2017 update: a report from the American Heart Association. Circulation. (2017) 135:e146–603. 10.1161/CIR.0000000000000485 28122885 PMC5408160

[4] Centers for Disease Control and Prevention Awareness of stroke warning symptoms–13 States and the District of Columbia, 2005. Morb Mortal Wkly Rep. (2008) 57:481 10.1001/jama.300.3.274 18463605

[5] Mettler FA Jr Bhargavan M Faulkner K Gilley DB Gray JE Ibbott GS Radiologic and nuclear medicine studies in the United States and worldwide: frequency, radiation dose, and comparison with other radiation sources–1950–2007 1. Radiology. (2009) 253:520–31. 10.1148/radiol.2532082010 19789227

[6] Takei Y Miyazaki O Matsubara K Shimada Y Muramatsu Y Akahane K. Nationwide survey of radiation exposure during pediatric computed tomography examinations and proposal of age-based diagnostic reference levels for Japan. Pediatr Radiol. (2016) 46:280–5. 10.1007/s00247-015-3474-x 26494635

[7] Thierfelder KM Sommer WH Baumann AB Klotz E Meinel FG Strobl FF. Whole-brain CT perfusion: reliability and reproducibility of volumetric perfusion deficit assessment in patients with acute ischemic stroke. Neuroradiology. (2013) 55:827–35. 10.1007/s00234-013-1179-0 23568701

[8] Cho G Kim JH Park TS Cho K Proposing a simple radiation scale for the public: radiation index. Nucl Eng Technol. (2017) 49:598–608. 10.1016/j.net.2016.10.005

[9] Wintermark M Lev M. FDA investigates the safety of brain perfusion CT. Am J Neuroradiol. (2010) 31:2–3. 10.3174/ajnr.A1967 19892810 PMC7964089

[10] Chodick G Bekiroglu N Hauptmann M Alexander BH Freedman DM Doody MM. Risk of cataract after exposure to low doses of ionizing radiation: a 20-year prospective cohort study among US radiologic technologists. Am J Epidemiol. (2008) 168:620–31. 10.1093/aje/kwn171 18664497 PMC2727195

[11] de González AB Mahesh M Kim KP Bhargavan M Lewis R Mettler F Projected cancer risks from computed tomographic scans performed in the United States in 2007. Arch Intern Med. (2009) 169:2071–7. 10.1001/archinternmed.2009.440 20008689 PMC6276814

[12] Journy NM Lee C Harbron RW McHugh K Pearce MS de González AB. Projected cancer risks potentially related to past, current, and future practices in paediatric CT in the United Kingdom, 1990–2020. Br J Cancer. (2017) 116:109. 10.1038/bjc.2016.351 27824812 PMC5220140

[13] Juluru K Shih J Raj A Comunale J Delaney H Greenberg E. Effects of increased image noise on image quality and quantitative interpretation in brain CT perfusion. Am J Neuroradiol. (2013) 34:1506–12. 10.3174/ajnr.A3448 23557960 PMC4108445

[14] Murphy A So A Lee TY Symons S Jakubovic R Zhang L. Low dose CT perfusion in acute ischemic stroke. Neuroradiology. (2014) 56:1055–62. 10.1007/s00234-014-1434-z 25252738

[15] Ng CS Chandler AG Wei W Anderson EF Herron DH Kurzrock R. Effect of sampling frequency on perfusion values in perfusion CT of lung tumors. Am J Roentgenol. (2013) 200:W155–62. 10.2214/AJR.12.8664 23345379 PMC3880201

[16] Othman AE Afat S Brockmann MA Nikoubashman O Brockmann C Nikolaou K. Radiation dose reduction in perfusion CT imaging of the brain: a review of the literature. J Neuroradiol. (2016) 43:1–5. 10.1016/j.neurad.2015.06.003 26452610

[17] Abels B Klotz E Tomandl B Villablanca J Kloska S Lell M. CT perfusion in acute ischemic stroke: a comparison of 2-second and 1-second temporal resolution. Am J Neuroradiol. (2011) 32:1632–9. 10.3174/ajnr.A2576 21816919 PMC7965399

[18] Yu L Fletcher JG Shiung M Thomas KB Matsumoto JM Zingula SN. Radiation dose reduction in pediatric body CT using iterative reconstruction and a novel image-based denoising method. Am J Roentgenol. (2015) 205:1026–37. 10.2214/AJR.14.14185 26496550 PMC4849891

[19] Yu Z Thibault JB Bouman CA Sauer KD Hsieh J. Fast model-based X-ray CT reconstruction using spatially nonhomogeneous ICD optimization. IEEE Trans Image Process. (2011) 20:161–75. 10.1109/TIP.2010.2058811 20643609

[20] Singh S Kalra MK Do S Thibault JB Pien H Connor OO. Comparison of hybrid and pure iterative reconstruction techniques with conventional filtered back projection: dose reduction potential in the abdomen. J Comput Assist Tomogr. (2012) 36:347–53. 10.1097/RCT.0b013e31824e639e 22592622

[21] Fang L Li S McNabb RP Nie Q Kuo AN Toth CA. Fast acquisition and reconstruction of optical coherence tomography images via sparse representation. IEEE Trans Med Imaging. (2013) 32:2034–49. 10.1109/TMI.2013.2271904 23846467 PMC4000559

[22] Li S Yin H Fang L. Group-sparse representation with dictionary learning for medical image denoising and fusion. IEEE Trans Biomed Eng. (2012) 59:3450–9. 10.1109/TBME.2012.2217493 22968202

[23] Huang J Zhang S Metaxas D. Efficient MR image reconstruction for compressed MR imaging. Med Image Anal. (2011) 15:670–9. 10.1016/j.media.2011.06.001 21742542

[24] Hamdan S Fukumizu Y Izumi T Yamauchi H Example-based face image super-resolution taking into consideration correspondence of facial parts. IEEE Trans Electr Electron Eng. (2017) 12:917–24. 10.1002/tee.22483

[25] Elad M Aharon M. Image denoising via sparse and redundant representations over learned dictionaries. IEEE Trans Image Process. (2006) 15:3736–45. 10.1109/TIP.2006.881969 17153947

[26] Trinh DH Luong M Dibos F Rocchisani JM Pham CD Nguyen TQ Novel example-based method for super-resolution and denoising of medical images. IEEE Trans Image Process. (2014) 23:1882–95. 10.1109/TIP.2014.2308422 24808354

[27] He K Zhang X Ren S Sun J Deep residual learning for image recognition. In: Proceedings of the IEEE Conference on Computer Vision and Pattern Recognition. Las Vegas, NV (2016). p. 770–8. 10.1109/CVPR.2016.90

[28] Erhan D Szegedy C Toshev A Anguelov D Scalable object detection using deep neural networks. In: Proceedings of the IEEE Conference on Computer Vision and Pattern Recognition. Columbus, OH (2014). p. 2147–54. 10.1109/CVPR.2014.276

[29] Burger HC Schuler CJ Harmeling S Image denoising: can plain neural networks compete with BM3D? In: IEEE Conference on Computer Vision and Pattern Recognition. Providence, RI: IEEE (2012). p. 2392–9. 10.1109/CVPR.2012.6247952

[30] Xie J Xu L Chen E Image denoising and inpainting with deep neural networks. In: Advances in Neural Information Processing Systems. Lake Tahoe (2012). p. 341–9.

[31] Kim J Kwon Lee J Mu Lee K Accurate image super-resolution using very deep convolutional networks. In: Proceedings of the IEEE Conference on Computer Vision and Pattern Recognition. Las Vegas (2016). p. 1646–54. 10.1109/CVPR.2016.182

[32] Bailer C Taetz B Stricker D. Flow fields: dense correspondence fields for highly accurate large displacement optical flow estimation. In: Proceedings of the IEEE International Conference on Computer Vision. Santiago (2015). p. 4015–23. 10.1109/ICCV.2015.457 30106705

[33] Kim S Bose NK Valenzuela H Recursive reconstruction of high resolution image from noisy undersampled multiframes. IEEE Trans Acoust Speech Signal Process. (1990) 38:1013–27. 10.1109/29.56062

[34] Aizawa K Komatsu T Saito T Acquisition of very high resolution images using stereo cameras. In: Visual Communications and Image Processing: Visual Communication. Vol. 1605 Boston, MA: International Society for Optics and Photonics (1991). p. 318–29.

[35] Park SC Park MK Kang MG Super-resolution image reconstruction: a technical overview. IEEE Signal Process Mag. (2003) 20:21–36. 10.1109/MSP.2003.1203207

[36] Nguyen N Milanfar P Golub G. A computationally efficient superresolution image reconstruction algorithm. IEEE Trans Image Process. (2001) 10:573–83. 10.1109/83.913592 18249647

[37] Irani M Peleg S Improving resolution by image registration. Graphical Models Image Process. (1991) 53:231–9. 10.1016/1049-9652(91)90045-L

[38] Stark H Oskoui P. High-resolution image recovery from image-plane arrays, using convex projections. JOSA A. (1989) 6:1715–26. 10.1364/JOSAA.6.001715 2585170

[39] Ng MK Shen H Lam EY Zhang L A total variation regularization based super-resolution reconstruction algorithm for digital video. EURASIP J Adv Signal Process. (2007) 2007:074585 10.1155/2007/74585

[40] Elad M Feuer A. Restoration of a single superresolution image from several blurred, noisy, and undersampled measured images. IEEE Trans Image Process. (1997) 6:1646–58. 10.1109/83.650118 18285235

[41] Dong C Loy CC He K Tang X Learning a deep convolutional network for image super-resolution. In: European Conference on Computer Vision. Zürich: Springer (2014). p. 184–99. 10.1007/978-3-319-10593-2_13

[42] Shi W Caballero J Huszár F Totz J Aitken AP Bishop R Real-time single image and video super-resolution using an efficient sub-pixel convolutional neural network. In: Proceedings of the IEEE Conference on Computer Vision and Pattern Recognition. Las Vegas (2016). p. 1874–83. 10.1109/CVPR.2016.207

[43] Oktay O Bai W Lee M Multi-input cardiac image super-resolution using convolutional neural networks. In: International Conference on Medical Image Computing and Computer-Assisted Intervention. Athens: Springer (2016). p. 246–54. 10.1007/978-3-319-46726-9_29

[44] Chen F Zhang L Yu H External patch prior guided internal clustering for image denoising. In: Proceedings of the IEEE International Conference on Computer Vision. Santiago (2015). p. 603–11. 10.1109/ICCV.2015.76

[45] Gu S Zhang L Zuo W Feng X Weighted nuclear norm minimization with application to image denoising. In: Proceedings of the IEEE Conference on Computer Vision and Pattern Recognition. Columbus, OH (2014). p. 2862–9. 10.1109/CVPR.2014.366

[46] Dabov K Foi A Katkovnik V Egiazarian K BM3D image denoising with shape-adaptive principal component analysis. In: Signal Processing with Adaptive Sparse Structured Representations. Saint Malo (2009).

[47] Zhang K Zuo W Chen Y Meng D Zhang L. Beyond a gaussian denoiser: residual learning of deep CNN for image denoising. IEEE Trans Image Process. (2017) 26:3142–55. 10.1515/9783110524116 28166495

[48] Mao XJ Shen C Yang YB Image restoration using convolutional auto-encoders with symmetric skip connections. arXiv [preprint]. arXiv:160608921 (2016).

[49] Baxes GA Digital Image Processing: Principles and Applications. New York, NY: Wiley (1994).

[50] Mendrik AM Vonken Ej van Ginneken B de Jong HW Riordan A van Seeters T. TIPS bilateral noise reduction in 4D CT perfusion scans produces high-quality cerebral blood flow maps. Phys Med Biol. (2011) 56:3857. 10.1088/0031-9155/56/13/008 21654042

[51] Salinas HM Fernández DC. Comparison of PDE-based nonlinear diffusion approaches for image enhancement and denoising in optical coherence tomography. IEEE Trans Med Imaging. (2007) 26:761–71. 10.1109/TMI.2006.887375 17679327

[52] Lin JW Laine AF Bergmann SR. Improving PET-based physiological quantification through methods of wavelet denoising. IEEE Trans Biomed Eng. (2001) 48:202–12. 10.1109/10.909641 11296876

[53] Fang R Zhang S Chen T Sanelli PC Robust low-dose CT perfusion deconvolution via tensor total-variation regularization. IEEE Trans Med Imaging. (2015) 34:1533–48. 10.1109/TMI.2015.2405015 25706579 PMC4779066

[54] Fang R Chen T Sanelli PC. Towards robust deconvolution of low-dose perfusion CT: Sparse perfusion deconvolution using online dictionary learning. Med Image Anal. (2013) 17:417–28. 10.1016/j.media.2013.02.005 23542422 PMC4196260

[55] Xiao Y Gupta A Sanelli PC Fang R STAR: spatio-temporal architecture for super-resolution in low-dose CT perfusion. In: International Workshop on Machine Learning in Medical Imaging. Springer (2017). p. 97–105. 10.1007/978-3-319-67389-9_12

[56] Szegedy C Liu W Jia Y Sermanet P Reed S Anguelov D Going deeper with convolutions. In: Proceedings of the IEEE Conference on Computer Vision and Pattern Recognition. Boston, MA (2015). p. 1–9. 10.1109/CVPR.2015.7298594

[57] Zeiler MD Fergus R Visualizing and understanding convolutional networks. In: European Conference on Computer Vision. Zürich: Springer (2014). p. 818–33. 10.1007/978-3-319-10590-1_53

[58] Jia Y Shelhamer E Donahue J Karayev S Long J Girshick R Caffe: convolutional architecture for fast feature embedding. In: Proceedings of the 22nd ACM International Conference on Multimedia. Orlando, FL: ACM (2014). p. 675–8. 10.1145/2647868.2654889

[59] Britten A Crotty M Kiremidjian H Grundy A Adam E. The addition of computer simulated noise to investigate radiation dose and image quality in images with spatial correlation of statistical noise: an example application to X-ray CT of the brain. Br J Radiol. (2004) 77:323–8. 10.1259/bjr/78576048 15107323

[60] Papyan V Elad M. Multi-scale patch-based image restoration. IEEE Trans Image Process. (2016) 25:249–61. 10.1109/TIP.2015.2499698 26571527

